# Awareness, treatment and control of type 2 diabetes among Chinese elderly and its changing trend for past decade

**DOI:** 10.1186/s12889-016-2874-7

**Published:** 2016-03-18

**Authors:** Miao Liu, Jianhua Wang, Yao He, Bin Jiang, Lei Wu, Yiyan Wang, Zhang Di, Jing Zeng

**Affiliations:** Institute of Geriatrics, Chinese PLA General Hospital, 28 Fuxing Road, Beijing, 100853 China; Beijing Key Laboratory of Aging and Geriatrics, Chinese PLA General Hospital, 28 Fuxing Road, Beijing, 100853 China; State Key Laboratory of Kidney Disease, Chinese PLA General Hospital, 28 Fuxing Road, Beijing, 100853 China; Department of Chinese Traditional Medicine and Acupuncture, Chinese PLA General Hospital, 28 Fuxing Road, Beijing, 100853 China

**Keywords:** Diabetes, Glycaemic control, Change, China, Elderly

## Abstract

**Background:**

This study aims to evaluate the awareness, treatment, control rate of type 2 diabetes and its risk factors among Chinese community elderly, and also examine the changing trend for the past decade.

**Methods:**

We conducted two population-based cross-sectional studies in a representative urban area of Beijing in 2001 and 2010 respectively, using with the same method. A total of 2,277 participants (943 male, 1,334 female) in 2001 and 2,102 participants (848 male, 1,254 female) in 2010 were recruited. All the participants diagnosed with diabetes were included in this study.

**Results:**

The prevalence of diabetes was 21.4 % and 24.8 % in 2001 and 2010 respectively. Among participants with diabetes, 74.2 % were aware of the condition, 51.0 % were treated, 20.1 % well controlled the condition in 2001, and the corresponding rates were 78.5 %, 69.3 %,15.9 % in 2010 respectively. Higher education level and a positive family history were related to better management of diabetes, while obesity and alcohol drinking showed a reverse direction.

**Conclusions:**

The prevalence and awareness of diabetes stayed high for the past decade. The treatment increased 18.3 % while the control rate decreased among community elderly for the past 10 years. It’s urgent to carry out effective measures to raise awareness, treatment, and control rate of diabetes in order to avoid growing disease burden in China.

## Background

With the population aging, huge socioeconomic change and abrupt transition of lifestyles, diabetes has become one of the most important global health problems, especially in low-and middle income countries [1, 2]. In 2014, there were 387 million people living with diabetes worldwide and the number keeps increasing [3]. In China, Gu [4] reported that the prevalence of diabetes was 5.5 % among 35–74 years old in 2003. Ning [5] reported the prevalence of diabetes had reached 11.6 % and the prevalence of impaired fasting blood glucose reached 50.1 % in 2010. The prevalence of diabetes in Beijing was also at a higher level. Monitoring data of adult chronic disease and risk factors in Beijing showed the prevalence of diabetes was 9.0 % in 2011 [6]. And previous studies showed that the prevalence increased from 5.2 in 2001 to 9.7 % in 2007 for urban residents, who might have a higher prevalence of diabetes [7]. The World Health Report indicates that diabetes is one major important cause of mortality in the world. Statistics from 2013 assessed 5,096,955 diabetes-related deaths in 20–79 year-olds in the world, of which 1,271,003 were recorded in China [8]. Also the direct economic burden of diabetes is huge, which has reached $9.1 billion, and has increased over time [9].

Several studies have published information on the prevalence of diabetes, and studies mainly from developing countries found the prevalence of diabetes is increasing while the awareness, treatment and control rate are quite low [10–12]. A multi-country study revealed that people in low-income countries and those with low socioeconomic status also have worse access to health care for timely diagnosis and treatment of non-communicable diseases than do those in high-income countries or those with higher socioeconomic status [13]. Previous studies from China also found that very few diabetic subjects had well controlled their condition [14, 15]. On the other side, as every separate study used different population and different criteria, it’s very hard to compare the results and assess the changes with time. The situation is similar in China, and little information was known about the changing trends of diabetes for the past decade, which has undergone a huge social-economic development and dramatic change in health related behaviors. Therefore, it’s urgent and important to examine the situation of diabetes and its changing trends in China for the past rapid development period. This study aims to solve the following questions: a. To identify the prevalence of diabetes in Chinese elderly and the absolute change during the past 10 years; b. To determine the awareness, treatment and control rate of diabetes and its changing trend in China for the past decade, and find the main risk factors.

## Methods

### Sampling method

A population-based cross-sectional study was conducted in Wanshoulu district, which was a representative urban area of Beijing. A two-stage clustering random sampling method was used to select a representative sample of community elderly. There are a total of 94 general residential communities in Wanshoulu district. We randomly selected 9 residential communities for the first step. Second, all households were chosen from the selected residents and only one eligible participant was randomly selected. Data from questionnaire, physical examination and a blood sample test were collected from the community elderly who were aged ≥ 60 years old. A total of 2680 residents aged 60–95 years old were invited to attend the survey, 2277 participants completed the survey and were eligible for analysis, with a response rate of 85.0 %. These elderly residents accounted for about 10 % of the total elderly population in the Wanshoulu district. Between 2009 and 2010, we did another survey in the same area using the same method. 2277 residents were invited and 2102 elderly residents completed the survey.

### Data collection

Each participant had a physical examination and answered a standardize questionnaire by specially trained doctors and nurses. The questionnaire included information on demographic factors, disease history, medical treatment, lifestyles and family history.

Weight of participants was measured in kilograms after removing with heavy clothes removed, and also height was measured in meters without shoes. Body mass index (BMI) was calculated as weight divided by height squared (kg/m^2^). Waist circumference (WC) was measured midway between the lowest rib and the iliac crest. Two blood pressures, with a 5-min interval, were obtained using the mercury sphygmomanometer in a sitting position after 30 min of rest, and the mean values were calculated. Data were collected at physical examination centers at Wanshoulu community health centers, near to the participants’ living place.

Overnight fasting venous blood samples were collected in vacuum tubes and were sent to the central certified laboratory of Chinese PLA general hospital within 30 min. Concentrations of total cholesterol (TC), triglycerides (TG), high-density lipoprotein cholesterol (HDL-C), low-density lipoprotein cholesterol (LDL-C) and fasting blood glucose (FBG) were assessed by colorimetric methods and commercially available reagents.

### Definitions

Since oral glucose tolerance test were not conducted in the field survey, only FBG was used in this study. Diabetes was defined as presence of one of the following conditions: FBG higher than 7.0 mmol/L; or been diagnosed as having diabetes before; or having received drug treatment for diabetes regularly.

The awareness of diabetes was defined as those who were aware of it among those with diabetes. The treatment of diabetes was defined as those who had taken medication for diabetes regularly or injected with insulin for diabetes. The control of diabetes was defined as those whose FBG level was less than 7.0 mmol/L. The treatment among awareness was defined as those who had taken medication for diabetes regularly or injected with insulin for diabetes among those who were aware of diabetes before. The control among treatment was defined as those whose FBG level was less than 7.0 mmol/L among those who had been treated.

### Statistical analysis

All data were double entered using Epidata 3.1 and the analysis was conducted using SPSS (19.0, No. of Serial: 5076595). *T* test for continuous variable and *χ*^2^ test for categorical variable were calculated by gender and by survey year. Age-specific prevalence, awareness, treatment and control of diabetes were calculated and *χ*^2^ was done for the difference during the past decade. Multivariate logistic regression was used to calculate odds ratios (ORs) and 95 % confidence interval (CI) for the potential factors. Two-tailed *p* < 0.05 was considered to be statistically significant.

### Ethical consideration

Ethics approval was obtained from the Ethics committee of Chinese PLA General Hospital. Each participant signed an informed consent prior to enrollment.

## Results

### Background Characteristics of the participants with and without diabetes

A total of 2,277 elderly participants (including 943 male and 1334 female) in 2001 year and 2,102 participants (including 848 male and 1,254 female) in 2010 completed the survey. Table 1 summarized the general characteristics of all participants. Compared with participants in 2001, participants in 2010 had older age, lower education level, lower smoking rate, less physical activity, higher drinking rate and a higher percentage of diabetes family history. The mean age was 67.9 ± 5.8 in 2001 and 71.2 ± 6.6 in 2010. Participants who had diabetes had a bigger WC, higher BMI, higher blood pressure, cholesterol and glucose levels. Also, compared with those without diabetes, participants with diabetes had a higher percentage of family history of diabetes. The prevalence of diabetes had an absolute increase of 3.4 % for the past decade, from 21.4 in 2001 to 24.8 % in 2010 (*p* = 0.030).

**Table 1:** General Characteristics in the Participants of Two Surveys at 2001 and 2010

Characteristic	2001				2010			
	diabetes (*n* = 487)	no diabetes (*n* = 1790)	*P*	Total (*n* = 2277)	diabetes (*n* = 521)	no diabetes (*n* = 1581)	*P*	Total (*n* = 2102)
Mean(SD)								
Age (yrs)	68.0 ± 5.6	67.9 ± 5.8	0.745	67.9 ± 5.8*	71.7(6.0)	71.0(6.8)	0.022	71.2 ± 6.6
WC (cm)	89.8 ± 8.7	87.1 ± 9.4	<0.001	87.7 ± 9.3	90.6(8.9)	87.3(8.9)	<0.001	88.3 ± 9.1
BMI (kg/m^2^)	26.1 ± 3.2	25.5 ± 3.5	<0.001	25.6 ± 3.5	25.6(3.3)	24.7(3.4)	<0.001	25.0 ± 3.4
SBP (mm Hg)	138.7 ± 20.8	136.7 ± 21.4	0.070	137.8 ± 21.3	142.9(20.3)	137.2(19.3)	<0.001	139.0 ± 19.8
DBP (mmHg)	76.5 ± 10.1	77.2 ± 10.5	0.191	77.1 ± 10.4	78.8(10.4)	76.7 (9.6)	<0.001	77.3 ± 9.9
TC (mmol/L)	5.3 ± 1.0	5.3 ± 1.8	0.842	5.3 ± 1.7	52(1.1)	5.3(1.0)	0.031	5.3 ± 1.0
TG (mmol/L)	1.9 ± 1.3	1.5 ± 0.3	<0.001	1.6 ± 1.1	1.8(1.0)	1.6(0.9)	<0.001	1.7 ± 0.9
HDL-C (mmol/L)	1.3 ± 0.6	1.4 ± 0.3	0.002	1.4 ± 0.4	1.3(0.3)	1.4(0.4)	<0.001	1.4 ± 0.4
LDL-C (mmol/L)	3.3 ± 0.8	3.3 ± 1.1	0.793	3.3 ± 1.0	3.3(0.9)	3.3(0.8)	0.098	3.3 ± 0.8
FPG (mmol/L)	8.5 ± 3.0	5.5 ± 0.6	<0.001	6.1 ± 1.9	7.6 (2.9)	5.4(0.5)	<0.001	6.1 ± 1.7
Number (%)								
Female	282(57.9)	1052(58.8)	0.731	1334(58.6)	388(59.5)	866(59.7)	0.926	1254(59.7)
Education > 7 years	277(56.9)	1037(57.9)	0.676	1314(57.7) *	458(70.2)	1064(73.4)	0.137	1522(72.4)
Married	414(85.0)	1498(83.7)	0.480	1912(84.0)	550(84.4)	1224(84.4)	0.973	1774(84.4)
Current smoking	66(13.6)	284(15.9)	0209	350(15.4) *	76(11.7)	155(10.7)	0.512	231(11.0)
Current alcohol drinking	72(14.8)	278(15.5)	0.698	350(15.4) *	154(23.6)	352(24.3)	0.745	506(24.1)
Physical exercise	353(72.5)	1327(74.1)	0.463	1380(73.8) *	549(84.2)	1255(86.6)	0.153	1804(85.8)
Family history of diabetes	71(14.6)	91(5.1)	<0.001	162(7.1) *	193(29.6)	179(12.3)	<0.001	372(17.7)

Data are mean ± SD for continuous values or n (%) for category values; *p < 0.05 for general characteristics of total participants of two surveys

### Awareness, treatment and control of diabetes by gender and age-group

The overall awareness rate increased from 74.2 in 2001 to 78.5 % in 2010 (Table 2), but the difference did not reach statistical significance (*p* = 0.106). The treatment rate had an absolute increase of 18.3 % (from 51.0 in 2001 to 69.3 % in 2010) for the past decade (*p* < 0.001), mainly because of the elderly aged under 80 years old. The control rate decreased from 20.1 in 2001 to 15.9 % in 2010 (*p* = 0.086). Among those who were aware of the diabetes, the treatment rate increased for an absolute change of 19.5 % (from 68.8 in 2001 to 88.3 % in 2010, *p* < 0.001). Among those who had taken drug regularly or injected with insulin, the control rate stayed stable for the two surveys (20.1 % in 2001 vs 18.8 % in 2010, *p* = 0.702).

**Table 2:** Awareness, treatment, control of diabetes by gender and age

		Male			Female			Total	
	2001	2010	*P*	2001	2010	*P*	2001	2010	*P*
Awareness									
60 ~ 69 years	89(71.2)	51(77.3)	0.367	131(75.3)	96(82.8)	0.131	220(73.6)	147(80.8)	0.072
70 ~ 79 years	53(73.6)	99(78.6)	0.427	74(75.5)	133(74.3)	0.825	1275(74.7)	232(76.1)	0.741
> 80 years	8(88.9)	19(95.0)	0.548	7(70.0)	11 (78.6)	0.633	15(78.9)	30(88.2)	0.365
Subtotal	150(72.8)	169(79.7)	0.097	212(75.2)	240(77.7)	0.475	362(74.2)	409(78.5)	0.106
Treatment									
60 ~ 69 years	57(45.6)	43(65.2)	0.010	89(51.1)	89(76.7)	<0.001	146(48.8)	132(72.5)	<0.001
70 ~ 79 years	36(50.0)	80(63.5)	0.064	54(55.1)	121(67.6)	0.039	90(52.9)	201(65.9)	0.005
> 80 years	7(77.8)	19(95.0)	0.159	6(60.0)	9(64.3)	0.831	13(68.4)	28(82.4)	0.245
Subtotal	100(48.5)	142(67.0)	<0.001	149(52.8)	219(70.9)	<0.001	249(51.0)	361(69.3)	<0.001
Control									
60 ~ 69 years	23(18.4)	7(10.6)	0.159	34(19.5)	24(20.7)	0.811	57(19.1)	31(17.0)	0.576
70 ~ 79 years	17(23.6)	21(16.7)	0.233	17(17.3)	23(12.8)	0.309	34(20.0)	44(14.4)	0.116
> 80 years	3(33.3)	4(20.0)	0.438	4(40.0)	4(28.6)	0.558	7(36.8)	6(23.5)	0.302
Subtotal	43(20.9)	32(15.0)	0.124	55(19.5)	51(16.5)	0.343	98(20.1)	83(15.9)	0.086
Treatment among awareness									
60 ~ 69 years	57(64.0)	43(84.3)	0.011	89(67.9)	89(92.7)	<0.001	146(66.4)	132(89.8)	<0.001
70 ~ 79 years	36(67.9)	80(80.8)	0.075	54(73.0)	121(91.0)	0.001	90(70.9)	201(86.6)	<0.001
> 80 years	7(87.5)	19(100.0)	0.116	6(85.7)	9(81.8)	0.829	13(86.7)	28(93.3)	0.459
Subtotal	100(66.7)	142(84.0)	<0.001	148(70.5)	219(93.2)	<0.001	249(68.8)	361(88.3)	<0.001
Control among treatment									
60 ~ 69 years	9(15.8)	5(11.6)	0.553	16(18.0)	22(24.7)	0.272	25(17.7)	27(20.5)	0.477
70 ~ 79 years	11(30.6)	14(17.5)	0.114	8(14.8)	20(16.5)	0.775	19(21.1)	34(16.9)	0.391
> 80 years	3(42.9)	4(21.1)	0.540	3(50.0)	3 (33.3)	0.914	6(46.2)	7(25.0)	0.320
Subtotal	232(23.0)	23(16.2)	0.184	27(18.1)	45(20.5)	0.565	50(20.1)	68(18.8)	0.702

### Risk factors and ORs of awareness, treatment and control rate of diabetes

Table 3 showed the univariate analysis of awareness, treatment and control rate of diabetes. The awareness rate was significantly lower among participants with lower education (0–6 education years) compared to those with higher education (≥7 education years) (68.6 % vs. 78.4 %), higher among those with family history of diabetes compared to those without (93.0 % vs. 71.0 %) in 2001. Similar to awareness, the treatment and control rate was also higher among those with higher education level and with family history. Overweight and obesity were reversely correlated with treatment and control rate. For example, the control rate of participants who were overweight or obesity was 19.1 %, which was significantly lower than those with lower BMI (23.8 %) in 2001. Also, participants who were current alcohol drinking had a lower treatment and control rate.

**Table 3:** Univariate analysis of awareness, treatment and control rate of diabetes (%)

		2001			2010	
	Awareness	Treatment	Control	Awareness	Treatment	Control
Age group						
60 ~ 69 years	73.6	48.8	19.4	80.8	72.5	17.6
70 ~ 79 years	74.7	52.9	20.0	76.1	65.9	14.4
> 80 years	78.9	68.4	36.8	88.2	82.4	23.5
Gender						
Male	72.8	48.5	21.4	79.7	67.0	15.1
Female	75.2	52.8	19.5	77.7	70.9	16.8
Education years						
0 ~ 6 years	68.6*	48.2*	23.7*	73.3*	65.2	14.9
> 7 years	78.4	54.8	15.7	80.8	71.1	16.7
Marital status						
Married	75.2	50.1	21.2	78.3	68.8	11.5
Not married	68.5	56.2	15.1	79.5	71.8	16.9
Current smoking						
Yes	71.2	48.5	25.8	74.2	68.2	13.6
No	74.6	51.4	19.4	79.1	69.5	16.5
Current alcohol drinking						
Yes	76.4	41.7	18.6*	72.9	59.5*	7.4*
No	73.7	52.5	30.6	78.5	72.3	18.8
Physical exercise						
Yes	73.7	51.4	19.2	78.6	68.6	15.9
No	75.4	50.0	23.1	77.8	72.8	17.3
BMI > 24 kg/m^2^						
Yes	72.1	49.7	19.1*	76.7	66.1*	14.1*
No	80.2	54.8	23.8	84.1	75.7	20.2
Family history of diabetes						
Yes	93.0*	74.6*	20.9	91.1*	74.0	17.8
No	71.0	47.0	16.9	63.3	67.0	15.3

*p < 0.05

Tables 4, 5 and 6 showed the multivariate logistic regression results. Two variables, education level and family history of diabetes, were statistically associated with awareness of diabetes. The corresponding ORs for education level and family history were 1.54 (95 % CI: 1.02–2.33), 5.33 (95 % CI: 2.09–13.57) in 2001 and 1.36 (95 % CI: 1.02–1.78), 4.17 (95 % CI: 3.47–5.06) in 2010 for awareness of diabetes.

**Table 4:** Multivariate logistic regression analyses on risk factors for awareness of diabetes among Chinese community elderly at two survey years

Variable	OR	95 % CI p
2001		
Education		0.039
> 7 years	1.54	1.02–2.33
0 ~ 6 years	1.00(Ref)	
Family history of diabetes		<0.001
Yes	5.33	2.09–13.57
No	1.00(Ref)	
2010		
Education		0.020
> 7 years	1.36	1.02–1.78
0 ~ 6 years	1.00(Ref)	
Family history of diabetes		<0.001
Yes	4.17	3.47–5.06
No	1.00(Ref)

**Table 5:** Multivariate logistic regression analyses on risk factors for treatment of diabetes among Chinese community elderly at two survey years

Variable	OR	95 % CI	*p*
2001			
Education			0.010
> 7 years	1.30	1.04–1.67	0.022
0 ~ 6 years	1.00(Ref)		
Family history of diabetes			<0.001
Yes	4.93	3.42–7.11	
No	1.00(Ref)		
2010			
BMI > 24 kg/m^2^			0.007
Yes	0.62	0.44–0.88	
No	1.00(Ref)		
Family history of diabetes			0.004
Yes	3.68	2.18–5.39	
No	1.00(Ref)		
Current alcohol drinking			0.044
Yes	0.69	0.47–0.99	
No	1.00(Ref)

**Table 6:** Multivariate logistic regression analyses on risk factors for control of diabetes among Chinese community elderly at two survey years

Variable	OR	95 % CI	*p*
2001			
BMI > 24 kg/m^2^			<0.001
Yes	0.54	0.44–0.67	
No	1.00(Ref)		
Education			0.003
> 7 years	1.54	1.16–2.05	0.97
0 ~ 6 years	1.00(Ref)		
2010			
BMI > 24 kg/m^2^			<0.001
Yes	0.53	0.43–0.65	
No	1.00(Ref)		
Education			0.009
> 7 years	1.38	1.08–1.75	0.97
0 ~ 6 years	1.00(Ref)		
Current alcohol drinking			0.043
Yes	0.62	0.39–0.99	
No	1.00(Ref)		

Family history was also related to treatment rate of diabetes. The corresponding ORs were 4.93 (95 % CI: 3.42–.7.11) in 2001 and 3.68 (95 % CI: 2.18–5.39) in 2010. Higher education was related to treatment rate of diabetes in 2001, which was in accordance with the awareness model. The OR was 1.30 (95 % CI: 1.04–1.67). In the model of data from 2010, the obesity and current alcohol drinking were also related to treatment rate, the ORs were 0.62 (95 % CI: 0.44–0.88) and 0.69 (95 % CI: 0.47–0.99). For control rate, two variables, higher education and obesity, were statistically associated with control rate of diabetes in both two years. Current alcohol drinking also had a significant effect on control rate of diabetes in 2010.

## Discussion

The present study reported the prevalence, awareness, treatment and control of diabetes and related risk factors among Chinese elderly and also reported the change for the past 10-years’ time. It revealed that in this Chinese community elderly, the prevalence of diabetes stayed high while the awareness, treatment and control rates were relatively low. And along with the time change, the treatment rate increased sustainably, while the awareness and control rate still stayed low for the past decade.

Diabetes is a rapidly growing threat to public health worldwide, especially in developing countries such as China [16]. Diabetes and related complications cause huge economic losses. In 2008, the direct medical cost reached $9.1 billion [17]. Considering the giant elderly in China and the huge disease burden caused by diabetes, it’s of critical importance to get the basic information about the prevalence and management status of diabetes among Chinese elderly and the related risk factors affecting the diabetes management. Early screening and treatment may lead to improvements in management and decrease the subsequent complications and related social and disease burden.

In this study, about 73.6 % of diabetic subjects were aware of their condition, 51.0 % received drug treatment, and 20.1 % well controlled their glucose level. The corresponding rates were 78.5 %, 69.3 % and 15.9 % 10 years later. The management situation of diabetes showed no optimism, especially the control rate. The results were similar to previous studies. Data from American National Health and Nutrition Examination Survey showed that the awareness, treatment, and control rates among U.S. elderly aged 65 and older elderly were 71.4 %, 50.9 % and 25.6 % respectively [12]. A cross-sectional national survey from Malaysia in 2001 showed that among elderly diabetic subjects in Malaysia, 65.2 % were aware of their status, 57.1 % had been treated and only 12.4 % had their diabetes controlled, which is slightly lower than our result [18]. Data from Korean National Health and Nutrition Examination Survey showed that the prevalence of diabetes increased from 16.4 to 20.3 % for elderly people, which was similar to our results [19]. A meta-analysis regarding the trends in prevalence, awareness, treatment, and control of diabetes mellitus in mainland China showed the pooled awareness, treatment, and control rate of diabetes were 45.81 %, 42.54 %, and 20.87 % for the general population. The study also showed that there was some fluctuation but no obvious increasing trend was observed for the management of diabetes from 1998 to 2012 [20], which are similar to our results.

Both univariate and multivariate analysis showed that higher education, positive family history and bad lifestyles were significantly related to the management of diabetes among Chinese elderly. Study among American elderly also showed that higher education is positively associated with awareness (OR = 1.30) and control (OR = 1.66) of diabetes [21]. This is similar to our results. Higher education was linked with better access and understanding of diabetes prevention related knowledge. Many studies have revealed that education level was related to better management and prognosis of chronic disease including diabetes [22, 23]. Some studies even revealed that better education would attenuate the relationship and outcomes of risk factor and disease. Also, family history was positively associated with management of diabetes in both two survey years in accordance with previous studies. African Americans with a family history of diabetes were more aware than those without, the corresponding relative risk was 1.09 (95 % CI: 1.03–1.15) [24]. Also, the study found obesity and alcohol drinking were reversely associated with management of diabetes. Previous studies have also demonstrated the conclusion. A study from US showed that obesity was associated with decreased diabetes awareness, and subjects with obesity had poorer glycemic controls compared to those without [25]. For alcohol drinking and management of diabetes, there were a certain of observational and experimental epidemiology studies revealed that unhealthy lifestyles were related with bad control of diabetes and corresponding interventions had huge effects.

The study had strict training process and quality assurance programs for both two surveys. Wanshoulu district is a representative metropolitan area of the geographic and economic characteristics in urban Beijing. The response rate was high and there was no statistically significant difference between participants who completed and those who didn’t complete the data. Second, our study showed that the management of diabetes among Chinese elderly is still grim. Although awareness and treatment rates increased for the past ten years, there were at least 30 % of diabetic subjects who were not aware of or not treated. The control rate decreased from 20.1 to 15.9 % in the past decade. There are studies who have revealed the prevalence and management of diabetes among the Chinese population, but they were of a different age group and time [4, 5, 14]. Few studies had assessed the situation among elderly which faces higher risk of having diabetes and also assess the changing trend for 10 years. Third, we also analyzed the related risk factors about management of diabetes. Results showed that higher education level, family history, obesity and alcohol drinking were related to management of diabetes, which gives us direction for better treatment and prevention.

This study also had several limitations need to be considered. First, the nature of cross-sectional study did not allow us to assess the temporal relationship between risk factors and management rates of diabetes. Further studies are needed to verify the conclusions. Second, oral glucose tolerance test were conducted due to field conditions, which may cause a missed diagnosis of potential diabetic subjects and low estimate of the prevalence of diabetes. But we used the same criteria in the both two surveys; this would have little influence on the changes of the prevalence of management rates. Third, the information of awareness and medication was self-reported by participants, there were potential reporting bias and recall bias. In order to decrease this bias, a standard questionnaire was used and all the investigators were trained before involving the field survey.

## Conclusions

In conclusion, according to the two population-based cross-sectional studies, the prevalence of diabetes was high and have been increasing in the past decade among Chinese elderly. The awareness and treatment rates were relatively low compared with developed countries, but had increased, especially the treatment rate. The control rate, which was less than 20.0 % of diabetic subjects, stayed low for 10 years. Considering the giant and growing elderly population and huge disease burden caused by diabetes, it’s urgent to improve the management of diabetes among Chinese elderly. A comprehensive strategy of early screening, optimal treating and proper public health strategies including health education and health promotion are needed, which will help improve the management of diabetes and also reduce the related disease burden.
